# Fast X-ray diffraction (XRD) tomography for enhanced identification of materials

**DOI:** 10.1038/s41598-022-23396-2

**Published:** 2022-11-09

**Authors:** Airidas Korolkovas

**Affiliations:** iTomography Corporation, Houston, TX 77021 USA

**Keywords:** Applied physics, Information theory and computation, Computational science, Software

## Abstract

X-ray computed tomography (CT) is a commercially established modality for imaging large objects like passenger luggage. CT can provide the density and the effective atomic number, which is not always sufficient to identify threats like explosives and narcotics, since they can have a similar composition to benign plastics, glass, or light metals. In these cases, X-ray diffraction (XRD) may be better suited to distinguish the threats. Unfortunately, the diffracted photon flux is typically much weaker than the transmitted one. Measurement of quality XRD data is therefore slower compared to CT, which is an economic challenge for potential customers like airports. In this article we numerically analyze a novel low-cost scanner design which captures CT and XRD signals simultaneously, and uses the least possible collimation to maximize the flux. To simulate a realistic instrument, we propose a forward model that includes the resolution-limiting effects of the polychromatic spectrum, the detector, and all the finite-size geometric factors. We then show how to reconstruct XRD patterns from a large phantom with multiple diffracting objects. We include a reasonable amount of photon counting noise (Poisson statistics), as well as measurement bias (incoherent scattering). Our XRD reconstruction adds material-specific information, albeit at a low resolution, to the already existing CT image, thus improving threat detection. Our theoretical model is implemented in GPU (Graphics Processing Unit) accelerated software which can be used to further optimize scanner designs for applications in security, healthcare, and manufacturing quality control.

## Introduction

### Literature overview

X-ray computed tomography (CT) is based on the measurement of X-ray transmission across a large region of interest (ROI), for example a suitcase in airport security screenings. After performing this measurement from multiple angles, it is possible to mathematically reconstruct the 3D density of the object. With multi-energy CT, we can also infer the average composition (effective atomic number) in 3D. Unfortunately for security applications, the density and atomic number of threat materials (drugs, explosives) can be very similar to that of harmless metals, ceramics, and plastics. A much more specific material fingerprint can be measured using X-ray diffraction (XRD). It is very sensitive to the spatial arrangement of atoms, which is highly distinct across thousands of different materials. XRD is especially well-suited for identifying crystals, as their periodic structure gives rise to very sharp diffraction peaks. This is advantageous for security screenings, since many threats are in fact crystals, crystalline powders, or semi-crystalline compound materials (crystal methamphetamine, cocaine and common explosives like TNT and RDX).

The ability to identify a material depends on the resolution of the reconstructed diffraction pattern. The resolution can be increased with tight collimation, both spatially and in terms of X-ray energy spectrum. The downside of collimation is the loss of photon flux^1^, which then requires long measurement times to compensate. A reasonable trade-off between resolution and flux is therefore key for an economically viable scanner. There is also the consideration of spatial resolution, but that is less important for security applications where the goal is to detect the most egregious threats like a suitcase loaded with pounds of an explosive or a drug. Small quantities of threats, or a multitude of different tiny threats within the same suitcase, is beyond the scope of this research. We therefore focus our attention on the resolution of the diffraction pattern, rather than the spatial resolution, unless explicitly stated otherwise.

An example of good resolution obtained in a real airport security setting is described in Ref.^2^. Their workflow consists of two machines: first a CT (computed tomography) scan to flag potential threat objects, and then a second pass through an X-ray diffractometer to provide a more specific material signature. To measure the XRD pattern of an object at high resolution, the authors have restricted the X-ray beam aperture to a thin pencil shape, as well as added collimators in front of their detector so that it accepts only a narrow range of scattering angles. Using an X-ray tube operating at 1.6 kW, the authors have scanned 4182 passenger baggage articles over the course of 53 days, or 3–4 items per hour. A similar two-stage system, XRD 3500, has been commercially deployed in several airports^3^. Also, another design using a cone-beam source has been patented by Halo technologies^4^. Notwithstanding these early success stories, further improvements in speed, cost, and accuracy are needed for XRD tomography to be adopted in commercial aviation on a wide scale.

The main avenue for increasing photon flux is to reduce the amount of collimation, although that leads to a loss of resolution of the measured diffraction pattern. The resolution can be partially recovered using computational techniques, which combine diffraction data taken at different X-ray energies, and from multiple source-detector positions with respect to the region of interest (ROI). A variety of mathematical and statistical approaches have been employed to reconstruct XRD patterns. For example, in the case of very low photon counts that are required for medical breast imaging, Ref.^5^ has demonstrated that Poisson likelihood maximization gives superior XRD reconstruction quality, compared to filtered back projection (FBP), whose main advantage in turn is computational speed^6^.

For XRD imaging, at least some amount of collimation seems to be inevitable, and various options have been discussed in literature, including delimitation, coded apertures, and combinations thereof. Ref.^7^ has used a fan-beam geometry, in conjunction with detectors collimated perpendicular to the fan plane. This geometry is sometimes called a “third generation XRD-CT”, and has been verified experimentally in Ref.^8^. Reconstruction of one slice results in a $(1+2)$-dimensional image, i.e. the one-dimensional diffraction pattern is resolved in a two-dimensional plane. This system has been patented, see Ref.^9^.

Reasonable XRD tomography speeds have been demonstrated even with thin pencil-beam sources, especially if they use the full polychromatic spectrum, and a fairly open coded aperture in front of the detector, see Ref.^10^. In Ref.^11^ the authors have even used a monochromatic pencil-beam, but the low flux was offset by combining CT and XRD reconstructions into a single edge-preserving algorithm, and forcing the diffraction patterns to be constant for each object in the phantom. In a subsequent study, Ref.^12^, the same authors have used a coded aperture instead of detector collimators, which has increased the flux, but decreased the resolution. To maintain acceptable resolution, the authors have emphasized the importance of image segmentation, as well as using multi-energy CT information to better account for attenuation along the diffracted beam pathways.

In Ref.^13^ the authors have simultaneously measured both CT and XRD signals, using a polychromatic fan beam. The energy-resolving detector was collimated to increase the resolution, at the expense of flux. The reconstructed XRD patterns have a reasonably high quality, although the paper does not mention the measurement time needed.

An even greater flux can be achieved by replacing detector collimators with a coded aperture, Ref.^14^. This study has only used two views per scan (snapshot tomography) and has imaged a single water vial. Alternatively, imaging time can be reduced by a factor of 6, if diffraction only needs to be reconstructed from a small region of the illuminated slice, see Ref.^15^. These examples with water show that XRD tomography is feasible not only for crystalline materials, but also for liquids. In Ref.^16^ the authors have reconstructed diffraction patterns of a phantom containing water, fat, collagen, and tricalcium phosphate (a substitute for bone). Such biology-inspired materials are amorphous rather than crystalline, hence their diffraction patterns are quite smooth. Nevertheless, XRD reconstruction was able to clearly distinguish between them.

Another type of aperture, a grating interferometer, was used to measure the dark-field image based on hard X-ray transmission^17^. Features with a higher scattering cross-section (bones), show up brighter than the weak scatterers (flesh), compared to a standard radiograph. At the expense of flux, image quality was improved over CT, but the results are not as material-specific as XRD.

Novel XRD tomography imaging modalities have been made possible by continuing improvements in X-ray tube technologies^18^, as well as the increasing availability of pixelated and energy-resolving detectors^19^. For explosives detection, Ref.^20^ gives a detailed introduction on coherent scatter computed tomography, energy-dispersive X-ray diffraction tomography, as well as Compton back-scatter imaging. When discussing future prospects, Ref.^21^ points to multi-focus X-ray sources (MFXS), where a single electron beam is deflected to multiple anodes, thus avoiding the need for moving parts, which should help with increasing scan speeds and decreasing maintenance costs. Reference^22^ showcases the importance of detector energy resolution. They have characterized their CdTe detector and found that its relative energy sensitivity is about 6% at momentum transfer of 2.5nm$^{-1}$. Multi-energy detectors are being actively developed, for example to be viable in case of high photon flux that can be an issue for transmission CT^23^. Whereas transmission CT operates in the high energy range of 30–180 keV, diffraction tomography would benefit from lower energies that result in greater spacing of diffraction peaks, and thus allow for higher resolution of the diffraction pattern. Furthermore, there is no risk of saturating the XRD detectors with high flux. Instead, better energy resolution would be highly welcome, since it directly impacts the XRD reconstruction quality.

Dedicated synchrotron facilities routinely use XRD tomography to obtain very high resolution measurements of various samples, for example polycrystal grains, see Ref.^24^. Such quality is enabled by the very high flux and intrinsic collimation of synchrontron X-ray beams. Additional focusing can be achieved with x-ray optics such as compounded refractive lenses (CRL) or Kirkpatrick–Baez (KB) mirrors, but they all require high vacuum and a lot of room, ${\mathcal{O}}(10\,{\text{m}})$, which are not readily available in airport security settings. XRD microtomography can reveal spatially resolved diffraction patterns of various engineering materials, for example concrete, see Ref.^25^. The resolution of their measurement is excellent both spatially and along the diffraction pattern axis. However, it used a very expensive particle accelerator (a synchrotron facility) and even then the acquisition has lasted 8 h.

So far, XRD tomography has not been commercially adopted on a large scale in security settings. The main obstacles are imaging time and cost. Nevertheless, XRD imaging is a worthwhile pursuit because of its unique ability to distinguish materials with a much higher specificity than any other modalities currently in use. Overall, there is a tradeoff between the speed, cost, and resolution of the system. Our aim in this theoretical paper is to strike a reasonable balance between these competing goals and to demonstrate how the overall design could be valuable in practice.

### Our XRD imaging proposition

In this article, we present an economically promising design for XRD tomography in airport security. A key requirement is to decrease the XRD scan time to be on par with the already widespread CT modality, while keeping any new hardware costs to a minimum. Due to these practical constraints, the resolution of XRD data will be limited, but still more informative than CT alone. With multi-energy imaging, CT can at most provide two numbers on each material, for example the density and the effective atomic number. If the XRD modality is capable to deliver at least one additional parameter per material, it would already lead to a significant improvement in threat detection capability. Here we are not concerned about the computational speed, as the price of computer hardware like GPUs (Graphics Processing Units) and cloud services continues to decrease, whereas the price of X-ray imaging components like sources and detectors is relatively fixed. In other words, we intend to rely heavily on computation, instead of expensive high-quality imaging hardware.

Borrowing from some of the previous designs, we shall use a polychromatic fan-beam and a 2D energy-resolving detector. A key novelty is that our detector will not have any delimitators, be it collimators or a coded aperture. To the best of our knowledge, this results in the highest achievable flux for XRD tomography. The diffracted photon counts will nevertheless be very low, which we shall address by performing image segmentation on the reconstructed CT data (which naturally has much higher counts). The purpose of segmentation is to reduce the number of unknowns for XRD reconstruction, by subdividing the suitcase into a small number of distinct materials. A similar idea was implemented in Ref.^12^, except that we remove the coded aperture, use a larger number of views, and install more detector pixels.

A suitcase will be illuminated slice-by-slice (in 2D), as it travels on a conveyor belt through the imaging plane. The computational workflow is as follows:

#### Step 1: Transmission (state-of-the-art)

Perform multi-energy computed tomography (MECT) reconstruction using X-ray transmission data. This step is widely used in airports and is available in real-time, see for example Ref.^26^.

#### Step 2: Segmentation (state-of-the-art)

Apply image segmentation to identify meaningful objects. This step is also widely used, see a review article in Ref.^27^. Furthermore, if multiple objects in the same suitcase have the same density and atomic number, we can assume that they are made from the same material, further reducing the number of unknowns for XRD reconstruction. The unknowns are the diffraction patterns of each unique material in the segmented image.

#### Step 3: Forward model (this article)

Using spatially resolved X-ray attenuation from MECT, as well as full knowledge of the scanner geometry and spectral properties, build a forward model for the expected XRD signal. This step is the key focus of this article and is presented in the next section.

#### Step 4: Reconstruction (state-of-the-art)

Reconstruct the XRD image, that is, find the diffraction pattern of each material that is most consistent with the observed data.

#### Step 5: Fingerprinting (to-be-done, details are likely proprietary and confidential)

Compare the reconstructed diffraction patterns against the database of known threat and benign materials. There are several databases available for a wide range of materials, including the International Centre for Diffraction Data (ICDD), the Inorganic Crystal Structure Database, and the Crystallography Open Database (COD). If the reconstructed diffraction pattern is consistent with any of the known threat materials, the suitcase is flagged for full inspection, such as manual search. Inputs other than XRD can be supplied for consideration (e.g., the size and shape of objects), as well as any other information about the passenger, flight, etc. available to the security operator.

## Method

### Outline

The novelty of this article is a detailed forward model of XRD image formation, which is Step 3 of the overall imaging workflow. We assume that Transmission (Step 1) and Segmentation (Step 2) have already been implemented. In addition, we assume that all relevant instrument parameters (geometry, source spectrum, detector response, etc.) are known. In this article, all inputs are known exactly, whereas with a real scanner there will be some imperfections, resulting in XRD quality degradation. It is common practice to apply various corrections (software and hardware based), as well as calibration procedures that mitigate the imperfections. It is beyond the scope of this article to assess how all of these effects may impact the XRD reconstruction quality.

Reconstruction and Fingerprinting (Steps 4 and 5) are also crucial stages in our threat detection workflow, and require further work, most notably gathering a large database of materials that are found in suitcases, as well as possible threats. In this article we only briefly touch on these two topics, just enough to show that threat detection with our design is in principle possible. More effort is needed in the post-processing domain to increase the commercial appeal of the scanner.

**Figure 1:**
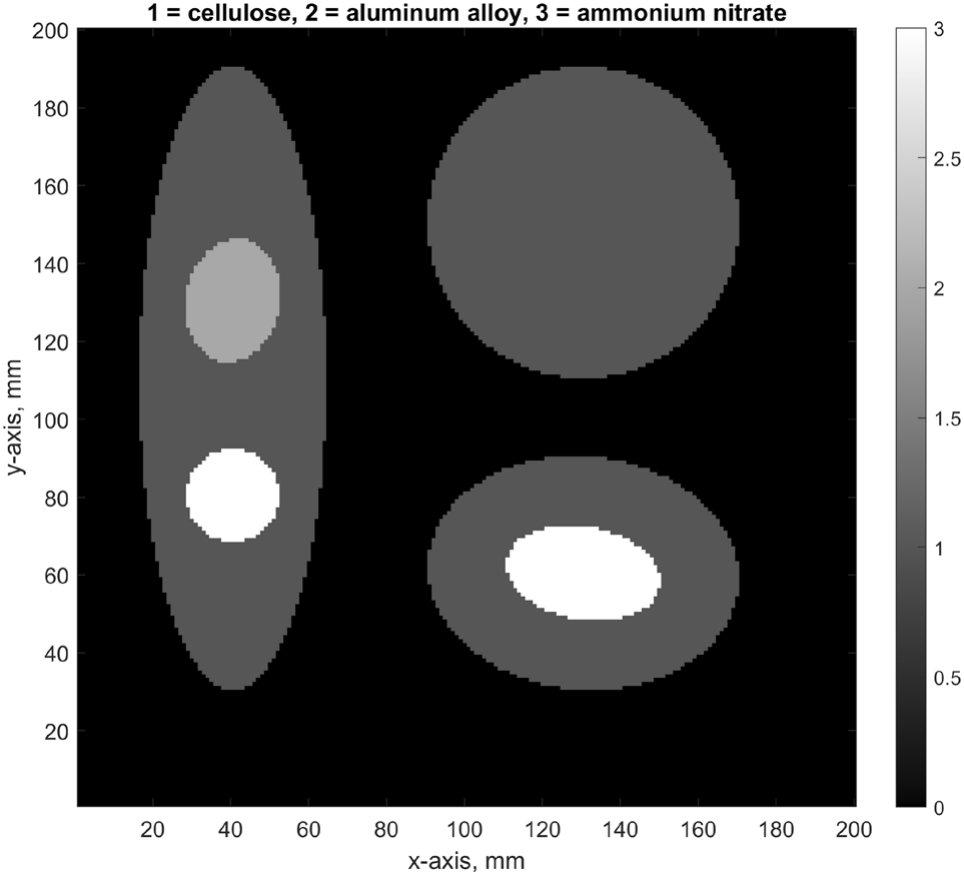
An XRD tomography image prototype. We show a 2D slice of a suitcase phantom that we assume was (1) reconstructed with MECT, (2) segmented into meaningful objects, and (3) grouped into distinct materials. Using this information and a simulated noisy XRD signal, we reconstruct the diffraction patterns for each unknown material and fingerprint them against a database of known materials. An XRD tomography image is therefore geometrically the same as MECT, but with a richer legend that assigns specific materials to each object, shown here by the color bar. The XRD-augmented picture is more informative than just the density and effective atomic number available from standard MECT.

**Figure 2:**
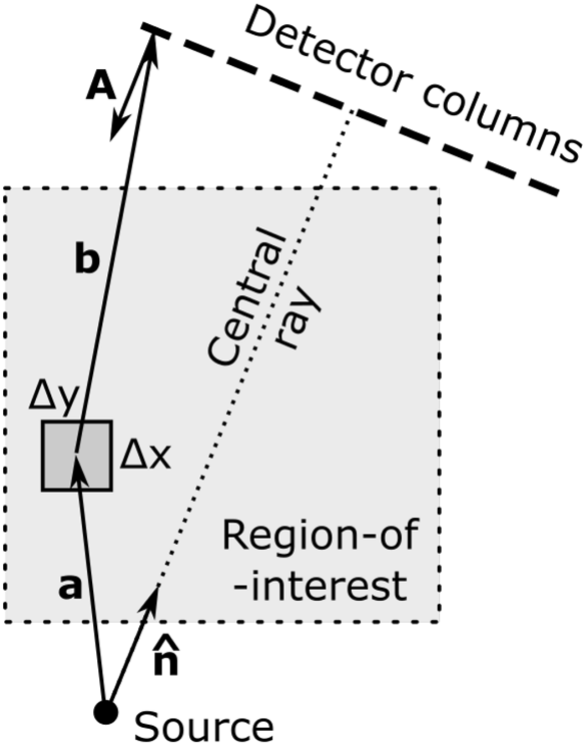
The top view of the scattering geometry. The symbols in the figure are: ${\textbf{a}} = \hbox{source-to-voxel vector}$, $b = \hbox{voxel-to-detector vector}$, $\Delta x$ and $\Delta y$ are the voxel dimensions in the horizontal plane. The anode plane that emits X-ray photons is oriented along the unit vector ${\hat{\textbf{n}}}$, whereas the detector pixel surface is given by the oriented surface area ${\textbf{A}}$ in units of mm$^2$. The ROI is fixed while the source-detector assembly rotates counterclockwise and records data at ${\text{SRC}}=32$ discrete angles.

**Figure 3:**
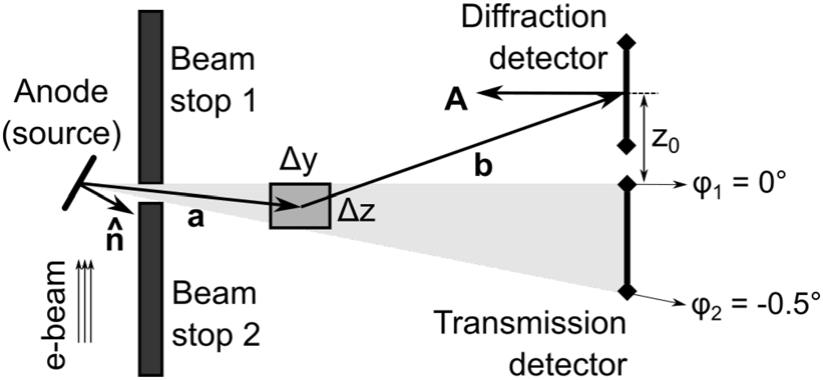
The side view of the scattering geometry. The symbols in the figure are: ${\textbf{a}} = \hbox{source-to-voxel vector}$, $b= \hbox{voxel-to-detector vector}$, $\Delta z$ is the voxel thickness along the tunnel (*z*) axis, $\Delta y$ is the in-plane width of the voxel. The anode plane that emits X-ray photons is oriented along the unit vector ${\hat{\textbf{n}}}$, whereas the detector pixel surface is given by the oriented surface area ${\textbf{A}}$ in units of $\hbox{mm}^2$. The first row of the diffraction detector panel is shown, with its center position at a height $z_0$ above the plane of the source. A similar figure is available in Ref.^12^, with a key difference being their 1D-scatter collimators that we remove entirely.

The starting point of our XRD forward model is shown in Fig. 1. The spatial resolution in this example is $200\times 200$ pixels (will be variable in reality, depends on MECT capabilities). The source and detector rotate around this region-of-interest (ROI) as shown in Fig. 2, although more complex arrangements like MFXS can also be accommodated. We only use a single 2D slice in this article (in a future development multiple slices should be combined to increase photon counts per material). The slice is defined by the thickness of the illuminated wedge (see Fig. 3), which is non-zero, so the 2D “pixels” will be henceforth referred to as voxels, even though there is just a single layer of them.

An XRD scan consists of a total of $M = {\text{SRC}}*{\text{COL}}*{\text{ROW}}*{\text{NRG}}$ measurements, which we label with a running index $m=1,2,\ldots ,M$. In this article we will show one example with ${\text{SRC}} = 32$ source positions (view angles), ${\text{COL}} = 1024$ detector columns, ${\text{ROW}} = 1$ detector rows, and ${\text{NRG}} = 64$ energy channels. MECT reconstruction provides the X-ray attenuation coefficient as a function of energy $\mu (E)$ at each of the voxels in Fig. 1. The only unknowns that we ask from an XRD scan are the values of the diffraction pattern as a function of wavevector transfer. In total there are $K = {\text{NWT}}*{\text{MAT}}$ unknowns, where ${\text{NWT}} = 256$ is our chosen number of wavevector transfer grid points, and ${\text{MAT}} = 3$ is the number of materials in the phantom. The individual unknowns are likewise labeled with a running index $k=1,2,\ldots ,K$. The relationship between the measured number of photons ${\textbf{N}} = N(m)$ and the unknowns ${\textbf{F}} = F(k)$ is linear (see Ref.^12^) and can be expressed in standard form of matrix multiplication with a column vector:

1 $${\mathbf{N}} = {\text{noise}}({\mathbb{A}} \cdot {\mathbf{F}} + {\text{bias}})$$

The dimensions of the model matrix ${\mathbb{A}}$ are [*M*, *K*], or $1.6\times 10^9$ elements in total. Each of these elements in turn contains contributions of every possible scattering pathway, which is the number of voxels multiplied by the number of energy channels, or $2.56\times 10^6$. Lastly, the likelihood of each scattering pathway is weighted by the photon survival probability due to attenuation, which is a line integral across the phantom, of a maximum length $200\sqrt{2}$, divided by the chosen step size, which in our case is 0.25 of a pixel. The grand total is in excess of $10^{18}$ (one quintillion) mathematical operations. We have implemented this computationally challenging task in GPU using CUDA (Compute Unified Device Architecture). A typical time for building the matrix on Titan V is approximately 1 h, but can vary a lot depending on the size and resolution of the phantom and the spectrum. There is room for substantial speedup by using more advanced algorithms than presented in this paper. One idea could be to calculate a series of sub-line integrals in a single go (a cumsum), rather than run a separate sum for each line integral. Furthermore, multi-GPU hardware and cloud computing are becoming very affordable and are a great match for our problem, since it separates naturally into parallel threads. In any case, once the matrix ${\mathbb{A}}$ is known, a full arsenal of linear algebra solvers, regularizers, neural nets etc. (see “Discussion” section) can be used to find the inverse ${\textbf{F}} = {\mathbb{A}}^{-1}\cdot {\textbf{N}}$, which reveals the diffraction patterns ${\textbf{F}}$, given the photon counts ${\textbf{N}}$. In this article we have used the Lucy–Richardson method, which generally works well in case of strong Poisson noise.

For each diffraction measurement *m*, we need to add the probabilities for all the possible ways that a photon could have traveled from the source to the detector. Because the source emits a wide fan-beam, photons can reach any voxel within the illuminated phantom slice, and from there they can diffract and reach any of the detector pixels. The phantom is large, so it is crucial to account for the X-ray attenuation on both the source-to-voxel and voxel-to-detector legs (the attenuation coefficients are known from MECT). Also, the beam is polychromatic and the detector is energy-sensitive, so we must sum the probabilities over the entire source spectrum and detector response to that spectrum. Mathematically, this paragraph can be summarized as a sum over all voxels and source energy channels:

2 $$\begin{aligned} N(m)&= \sum _{{\text{vox}}=1}^{{\text{VOX}}} \sum _{{\text{nrgsrc}}=1}^{{\text{NRG}}} (G = {\text{geometry}})\times (\eta = {\text{spectrum}})\times (P_{{\text{in}}} P_{{\text{out}}} = {\text{attenuation}})\nonumber \times (F ={\text{cross-section}}) \end{aligned}$$

In more detail, the factors are:

1. Geometrical factor *G* includes trigonometry, solid angles, etc. We compute it in “Source trajectory” and “Scattering geometry” sections.
2. Source spectrum and detector response to it $\eta$, elaborated in “Energy spectrum and detector response” section.
3. The incoming photon survival probability $P_{{\text{in}}}$ from the source to the voxel, and the outgoing photon survival probability $P_{{\text{out}}}$ from the voxel to the detector. They both depend on the X-ray attenuation $\mu (E)$ that is known from a prior MECT reconstruction and we show how to compute them in “Beam attenuation” section.
4. Differential scattering cross-section per unit volume *F*(*k*) (depends on the material and a combination of X-ray energy as well as the scattering angle). In “Diffraction patterns” section we show how to use diffraction patterns found in literature to generate the ground truth in our imaging workflow.

The last factor is the differential scattering cross-section *F*(*k*), which is the column of unknowns for the purpose of XRD reconstruction. However, we will also need to simulate ground-truth measurement data, in which case *F*(*k*) is known and is associated with each of the materials that we choose to add to our phantom. We note that for simpler materials like pure crystalline powders the differential scattering cross-section is proportional to the structure factor of the material, up to a multiplicative constant (which involves the density, the Thomson electron radius, etc.). In general, for composite materials that are found in modern explosives, the differential scattering cross-section is a mixture of various molecular structure factors, that may also interfere non-linearly. Trying to resolve the molecular structure factors of various components that make up explosives is beyond the scope of this article, and we will instead stick with the differential scattering cross-section that characterizes the material as a whole.

As we shall see in “Diffraction patterns”, diffraction patterns of typical crystals have very sharp peaks, which change rapidly across a single voxel, and across a single energy channel. A naïve solution would be to use finer grids for the phantom and the spectrum, but that is cost-prohibitive since even with the current coarse model we are already at $10^{18}$ operations. Furthermore, we would need to subdivide the detector pixels as well as the source focal spot into smaller areas, which would render the computation practically unfeasible. Our solution to this “curse of dimensionality” is to smear the high-resolution diffraction pattern with a low-pass filter that has the width of each coarse diffraction pathway, before running Eq. (2). “A small, but finite scattering pathway” section shows how to compute the width of any given pathway. Lastly, “The forward projector and model matrix” section shows how to factor out the unknowns *F*(*k*), by intersecting the diffraction pathway width with the width of the reconstruction bin, thus isolating the matrix coefficients ${\mathbb{A}}(m,k)$.

The final “Compton scattering background” section discusses a known source of bias, specifically incoherent Compton scattering. It is possible to estimate and correct for this bias, which would otherwise degrade the XRD reconstruction quality.

### Source trajectory

Security scanners can use either a rotating source-detector assembly, or a stationary multi-focus source (MFXS) arrangement, for example an irregular polygon inscribed within the available tunnel space. Our theoretical model can be adapted to either choice. In this article, we only consider a rotating source, which has a circular trajectory. Likewise, the detector could have irregular or curved shape as well, but here we only show an example with a flat panel detector. The region-of-interest (ROI) is a square of size $L_x = L_y = {200}\,\hbox{mm}$. We assume that the phantom has constant composition along the third axis ${\hat{\textbf{z}}} = {\textbf{z}}/|{\textbf{z}}|$ (unit vector), which is justified if the illuminated fan beam is thinner than the objects in the phantom. For computational convenience, the origin of the Cartesian coordinate system is chosen in the left bottom corner of the ROI. The plane in which the source rotates defines the origin of the *z*-axis. The trajectory of the source is thus given by

3 $$\begin{aligned} {\textbf{R}}_{{\text{src}}} = (L_x/2 + \rho _{{\text{src}}}\sin \alpha ) {\hat{\textbf{x}}} + (L_y/2 - \rho _{{\text{src}}}\cos \alpha ){\hat{\textbf{y}}} \end{aligned}$$

where $\rho _{{\text{src}}} = 150\,\hbox{mm}$ is the distance from the source to the middle of the ROI and $\alpha = 2\pi ({\text{src}}/{\text{SRC}})$ is the view angle, with ${\text{src}} = 0,1,\ldots ,({\text{SRC}}-1)$ being the source index. The detector trajectory is given by

4 $$\begin{aligned} {\textbf{R}}_{\text{det}} = (L_x/2 - \rho _{\text{det}}\sin \alpha + c\cos \alpha ) {\hat{\textbf{x}}} + (L_y/2 + \rho _{{\text{det}}}\cos \alpha + c\sin \alpha ){\hat{\textbf{y}}} + z_0 {\hat{\textbf{z}}} \end{aligned}$$

where $c = ({\text{col}} - {\text{COL}}/2 + 0.5)*({\text{column pitch}})$ is the position of the detector column with respect to the central ray. The radius of the detector trajectory is $\rho _{\mathrm{\text{det}}} = 170\,\hbox{mm}$. In this work the detector column pitch is chosen to be 0.5 mm, while the height of the first detector row is $z_0 = 10\,\hbox{mm}$ (see Fig. 3). This offset is required because in reality, due to the finite source size, a small fraction of the transmitted beam can protrude above the $z=0$ plane, hence the first row must be high enough to avoid it. At the same time, diffraction detectors should be as close to the direct beam as possible, to capture the smallest scattering angles where many XRD peaks can often be found.

The vector normal to the detector surface area is given by

5 $$\begin{aligned} {\hat{\textbf{A}}} = {\hat{\textbf{x}}} \sin \alpha - {\hat{\textbf{y}}} \cos \alpha \end{aligned}$$

whereas the source surface area (the anode) is perpendicular to

6 $$\begin{aligned} {\hat{\textbf{n}}} = -{\hat{\textbf{x}}} \sin \alpha \sin \beta + {\hat{\textbf{y}}} \cos \alpha \sin \beta - {\hat{\textbf{z}}} \cos \beta \end{aligned}$$

where $\beta = {30}^\circ$ is the tilt of the anode plane.

### Scattering geometry

The geometry factor *G* in Eq. (2) is composed of the following terms:

7 $$\begin{aligned} G = \underbrace{\Delta x \Delta y \Delta z}_{{\text{volume}}} \underbrace{|{\textbf{a}}|^{-2}}_{{\text{fluence}}} \underbrace{\left( 1+\cos ^2 \theta \right) /2}_{{\text{polarization}}} \underbrace{\Delta \Omega }_{{\text{solid angle}}} \end{aligned}$$

There is one such factor for every scattering pathway, which is a triangle between the source, the voxel, and the detector, as shown in Figs. 2 and 3. The source-to-voxel vector is ${\textbf{a}}$ and the voxel-to-detector vector is ${\textbf{b}}$. The in-plane area of the voxel $\Delta x\Delta y$ is determined by the spatial resolution of the preceding MECT reconstruction. In this work we assume that MECT image is available and has a resolution of $\Delta x = \Delta y =1\,\hbox{mm}$.

The illuminated volume (see gray area in Fig. 3) has the shape of a wedge, and is controlled by the beamstop opening. The top of the illuminated wedge is set at $\varphi _1 = {0}^\circ$, while the bottom is slanted at an angle $\varphi _2 = {-0.5}^\circ$ (both tunable settings). The thickness of a particular voxel is thus

8 $$\begin{aligned} \Delta z = \left( \frac{a_x n_x + a_y n_y}{\sqrt{n_x^2 + n_y^2}}\right) (\tan \varphi _1 - \tan \varphi _2) \end{aligned}$$

where $a_x$ and $a_y$ are the in-plane cartesian coordinates of the source-to-voxel vector ${\textbf{a}}$. The position of the middle of the voxel along the *z*-axis is:

9 $$\begin{aligned} v_z = \left( \frac{a_x n_x + a_y n_y}{\sqrt{n_x^2 + n_y^2}}\right) \left( \frac{\tan \varphi _1 + \tan \varphi _2}{2}\right) \end{aligned}$$

This position is used to compute the *z* components of both ${\textbf{a}}$ and ${\textbf{b}}$. The inverse square length $|{\textbf{a}}|^{-2}$ accounts for the photon fluence (number of photons per surface area) at the location of the voxel. Notice that the voxel thickness, Eq. (8), increases linearly with the source-to-voxel distance. Together with the $|{\textbf{a}}|^{-2}$ fluence factor, the number of diffracted photons goes down as $|{\textbf{a}}| ^{-1}$.

The angle between the two legs is $\cos \theta = {\hat{\textbf{a}}} \cdot {\hat{\textbf{b}}}$. It is used to compute the polarization factor $\left( 1+\cos ^2\theta \right) /2$, which applies for non-polarized X-ray sources like vacuum tubes^28^. Finally, from the vantage point of the voxel, a detector of oriented surface area ${\textbf{A}}$ subtends a solid angle

10 $$\begin{aligned} \Delta \Omega = \frac{ {\textbf{A}}\cdot {\textbf{b}}}{|{\textbf{b}}|^3} \end{aligned}$$

The overall units of the geometry factor *G* are mm. In this section we have assumed that the phantom is uniform over a thickness of at least $\Delta z$.

### Energy spectrum and detector response

Transmission tomography usually operates with hard X-rays in the 30–180 keV range, which have weak attenuation needed to penetrate thick materials. X-ray diffraction, on the other hand, is typically done on very small samples in a laboratory setting, and using much softer X-rays, for example 8.04 keV which is the copper K-$\alpha$ line. For small samples the beam attenuation is not important, and a lower X-ray energy is preferred as it broadens the spacing of diffraction peaks seen on the detector (see Eq. (31)), hence increasing the resolution. Our combined transmission $+$ diffraction scanner should strike some middle ground between these two extremes.

**Figure 4:**
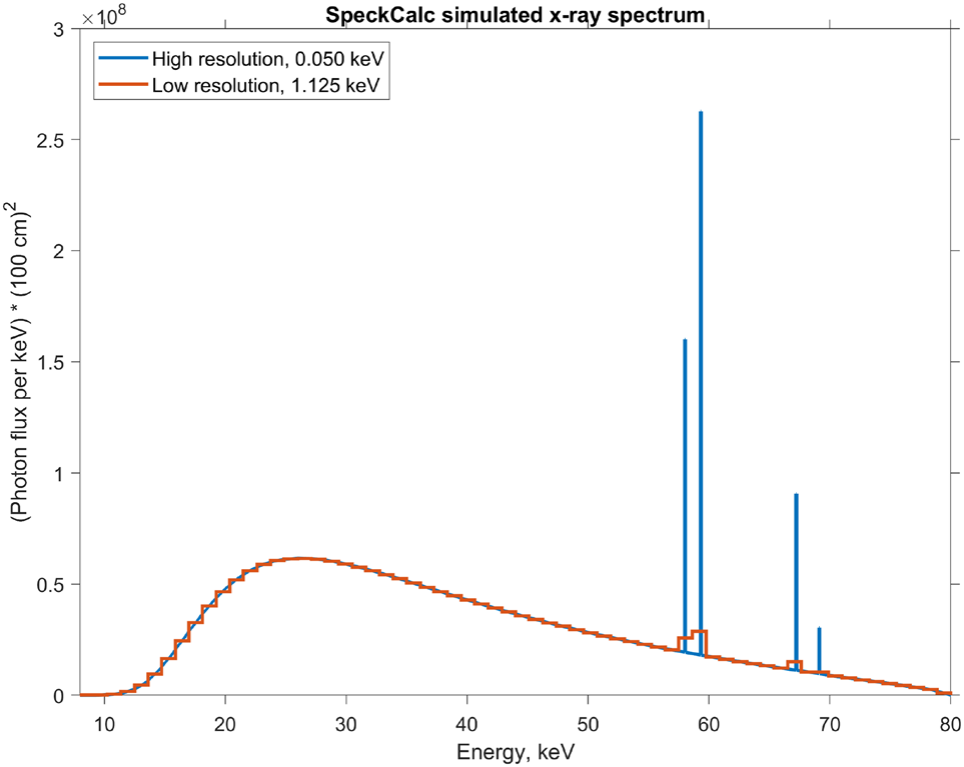
Source spectrum simulation from SpekCalc. The number of photons is proportional to the source current and exposure time, which we have assumed to be 10 mA and 0.1 ms, or 1 mC of electron charge.

The optimal spectrum will depend on the specific application, so in this paper we just pick a reasonable value of 80 keV for the anode voltage and a default 1 mm aluminum filter to remove the lowest energy photons. The spectrum values $\Phi (E)$ provided by SpekCalc (Fig. 4) refer to the number of photons per $\hbox{cm}^{2}$ at a reference distance $a_0 = {100}\,\hbox{cm}$, per energy bin of width 1 keV. The absolute number of photons is proportional to the time of exposure which we set to 0.1ms per source per *z*-slice, and to the source current which we set to 10 mA, but these settings can vary wildly between different applications of XRD imaging. Anisotropic features of the spectrum such as the heel effect are not considered in this work, but would be straightforward to implement within the current algorithm.

This high-resolution spectrum will never be observed with a realistic energy-sensitive detector, which has a resolution of roughly

11 $$\begin{aligned} \sigma = (1.61\,{\hbox{keV}} + 0.025E_{{\text{src}}})/2 \end{aligned}$$

as detailed in experimental^29^ and theoretical^30^ studies. Quite a few years have passed since the publication of these papers, hence we have taken the liberty to assume that the energy resolution has been improved by a factor of two, or will be so in the near future. We discretize the spectrum to a reasonable number of ${\text{NRG}} = 64$ energy bins, spaced equidistantly between $E_{{\text{min}}} = 8\,{\hbox{keV}}$ and $E_{{\text{max}}} = 80\,{\hbox{keV}}$, with a bin width $\Delta E = (E_{{\text{max}}} - E_{{\text{min}}})/{\text{NRG}} = 1.125\,\hbox{keV}$, which is on the lower end of the energy resolution range $\sigma = 0.905-1.805\,\hbox{keV}$. The source energy bins are labeled with the index ${\text{nrgsrc}} = 1,2,\ldots ,{\text{NRG}}$, and similarly the detector energy bins are labeled with the index ${\text{nrgdet}} = 1,2,\ldots ,{\text{NRG}}$. We can then compute the efficiency of capturing a source photon from the bin nrgsrc at a detector bin nrgdet:

12 $$\begin{aligned} \eta ({\text{nrgsrc}}, {\text{nrgdet}}) = \int _{E_1}^{E_2} dE_{{\text{src}}} \int _{E_3}^{E_4} dE_{{\text{det}}} \frac{\Phi (E_{\text{src}})a_0^2}{\sqrt{2\pi \sigma ^2}}\exp \left( -\frac{(E_{\text{src}} - E_{\text{det}})^2}{2\sigma ^2}\right) \end{aligned}$$

The limits of integration (the bin edges) are given by

13a $$\begin{aligned} E_1= E_{\text{min}} + (\text{nrgsrc}-1)\Delta E \end{aligned}$$

13b $$\begin{aligned} E_2= E_{\text{min}} + \text{nrgsrc}\Delta E \end{aligned}$$

13c $$\begin{aligned} E_3= E_{\text{min}} + (\text{nrgdet}-1)\Delta E \end{aligned}$$

13d $$\begin{aligned} E_4= E_{\text{min}} + \text{nrgdet}\Delta E \end{aligned}$$

**Figure 5:**
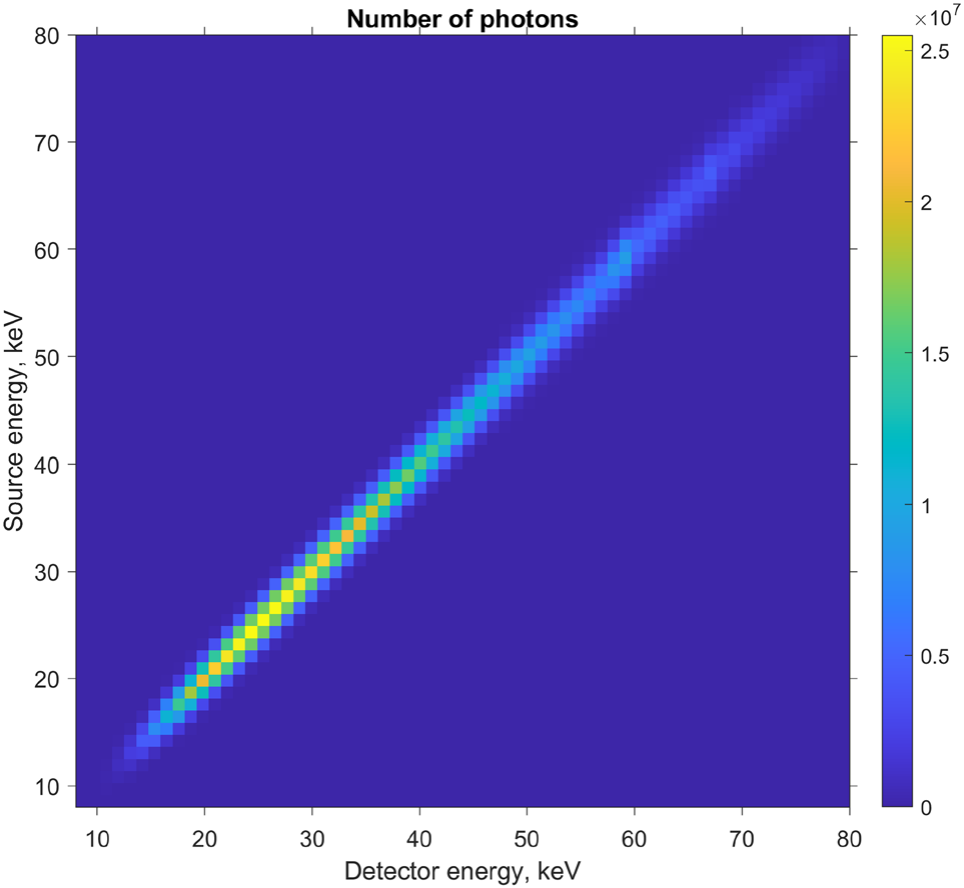
Convolution of detector response with the source spectrum. Due to rapid drop-off away from the diagonal, we use only 5 bins to the left and right to sum over the spectrum (see Eq. (2)), hence 11 terms in total, rather than all 64.

The result of Eq. (12) is a dimensionless number of photons and is shown in Fig. 5. In other words, $\eta (\text{nrgsrc}, \text{nrgdet})$ is the convolution of the source spectrum and the detector resolution, for all bin pairs. We have assumed the detector gain to be one, although in reality it should be calibrated, pixel-by-pixel, and the results incorporated to Eq. (12).

### Beam attenuation

The phantom used in this work is shown in Fig. 1, and corresponds to the gray area in Fig. 2. It consists of $(\text{NX}=200) \times (\text{NY} = 200)$ voxels of size 1 mm$^2$. We assume that multi-energy computed tomography (MECT) has been performed for this phantom, using the transmission detector data, see the transmission detectors in Fig. 3. The result of MECT is X-ray attenuation coefficient as a function of energy at each point

14 $$\begin{aligned} {\textbf{r}} = [(\text{nx}-0.5)\Delta x, (\text{ny}-0.5)\Delta y] \end{aligned}$$

of the phantom. Reference^31^ shows that X-ray attenuation can be well approximated as a sum of photoelectric ($a_1$) and Compton ($a_2$) coefficients:

15 $$\begin{aligned} \mu ({\textbf{r}}, E) = a_1({\textbf{r}}) f_1(E) + a_2({\textbf{r}}) f_2(E) \end{aligned}$$

The energy basis functions are given by

16a $$\begin{aligned} f_1= \varepsilon ^{-3} \end{aligned}$$

16b $$\begin{aligned} f_2= \frac{1+\varepsilon }{\varepsilon ^2} \left[ \frac{2(1+\varepsilon )}{1+2\varepsilon } - \frac{\log (1+2\varepsilon )}{\varepsilon }\right] + \frac{\log (1+2\varepsilon )}{2\varepsilon } - \frac{1+3\varepsilon }{(1+2\varepsilon )^2} \end{aligned}$$

where $\varepsilon = E/m_ec^2$ is the dimensionless photon energy (i.e., divided by the electron rest energy $m_ec^2$). In this paper we make an optimistic assumption that MECT reconstruction is ideal, in which case the coefficients for each material should be the same as

17a $$\begin{aligned} a_1= K_1 \rho \frac{N_1 Z_1^n + N_2 Z_2^n + \cdots }{N_1 M_1 + N_2 M_2 + \cdots } \end{aligned}$$

17b $$\begin{aligned} a_2= K_2 \rho \frac{N_1 Z_1 + N_2 Z_2 + \cdots }{N_1 M_1 + N_2 M_2 + \cdots } \end{aligned}$$

Here $n=4.2$ and $K_1 = 1.047\times 10^{-7}\,\hbox{cm}^{2}/\hbox{mol}$ are empirical parameters, while $K_2 = 2\pi r_e^2 N_A = 0.30\,\hbox{cm}^2/\hbox{mol}$ is the Compton parameter that can be computed from the classical electron radius $r_e$ and the Avogadro’s number $N_A$. Other symbols are:

- $\rho$ is the mass density of the material in g/cm$^{3}$,
- $N_{1,2,\ldots }$ is the number of atoms,
- $Z_{1,2,\ldots }$ is the atomic number, and
- $M_{1,2,\ldots }$ is the atomic weight.

For example, the chemical composition of ammonium nitrate is $\text{NH}_4\text{NO}_3$, which in our notation is $Z=[1,7,8]$, $N=[4,2,3]$, and $M = [1.00784,\,14.0067,\,15.999]\,\hbox{g mol}^{-1}$.

The photon survival probabilities mentioned in Eq. (2) are given by:

18a $$\begin{aligned} P_{\text{in}}(E)= \exp \left(-f_1(E)\int _{\text{src}}^{\text{vox}} a_1({\textbf{r}})\; dr - f_2(E)\int _{\text{src}}^{\text{vox}} a_2({\textbf{r}})\; dr \right) \end{aligned}$$

18b $$\begin{aligned} P_{\text{out}}(E)= \exp \left(-f_1(E)\int _{\text{vox}}^{\text{det}} a_1({\textbf{r}})\; dr - f_2(E)\int _{\text{vox}}^{\text{det}} a_2({\textbf{r}})\; dr\right) \end{aligned}$$

In the simulation, the line integrals are replaced by discrete sums with step size $\Delta r = 0.25$ of the voxel size (which is our internal length unit defined as one, i.e., $\Delta x = \Delta y = 1$, and all the other lengths are relative to this). In general, the query points along the lines do not match with the locations of the voxels which are on a Cartesian grid, hence we fetch the values for (${a}_1({\textbf{r}})$, ${a}_2({\textbf{r}})$) using bilinear interpolation, and assume them to be zero outside of the region-of-interest (ROI).

### Diffraction patterns

The vast majority of XRD studies report the scattered intensity in arbitrary units. This is adequate for determining crystal structures, which can be inferred solely from the positions and relative intensities of the diffraction peaks. On the other hand, a key consideration for an XRD imaging system is the photon flux, which in turn depends on the scattering cross-sections in absolute units. This step is crucial to design a system that strikes a fair balance between the flux and the accuracy of the wavevector transfer *q*, which is needed to resolve individual XRD peaks. Currently, intensity calibration is experimentally feasible only for a limited number of situations. In particular, small angle X-ray scattering (SAXS) machines are sometimes calibrated to an absolute scale, since in that geometry the Ewald sphere can be assumed as flat and the detector plane can be perfectly matched with it. In wide angle X-ray scattering (WAXS), this assumption is not applicable and an additional correction is required for the angle it makes with the tangent of the sphere surface. Due to this and other complications, most authors do not attempt to calibrate their WAXS data, let alone XRD. At present, there exist several studies performed using the same sample on both SAXS and WAXS machines having an overlap in their *q*-ranges, which allows a straightforward cross-calibration of the two datasets. Available examples include silver behenate^33^, NiBpene flexible MOFs (metal-organic frameworks)^34^, and isotactic polypropylene^35,36^.

For general materials of interest to security, healthcare, and manufacturing, absolute XRD intensity calibration is not readily available. Nevertheless, we can roughly predict the background level of scattering, using theoretical Rayleigh and Compton functions *R*(*q*) and *C*(*q*), which are tabulated for each atom of the periodic table in Ref.^37^. For materials that contain multiple atomic species, the differential scattering cross-section per unit volume is:

19 $$\begin{aligned} \left( \frac{1}{V}\frac{d\sigma }{d\Omega }\right) _{\text{R}}= r_e^2 N_A \rho \left( \frac{N_1 R_1^2 + N_2 R_2^2 + \cdots }{N_1 M_1 + N_2 M_2 + \cdots }\right) \end{aligned}$$

20 $$\begin{aligned} \left( \frac{1}{V}\frac{d\sigma }{d\Omega }\right) _{\text{C}}= r_e^2 N_A \rho \left( \frac{N_1 C_1 + N_2 C_2 + \cdots }{N_1 M_1 + N_2 M_2 + \cdots }\right) \end{aligned}$$

**Figure 6:**
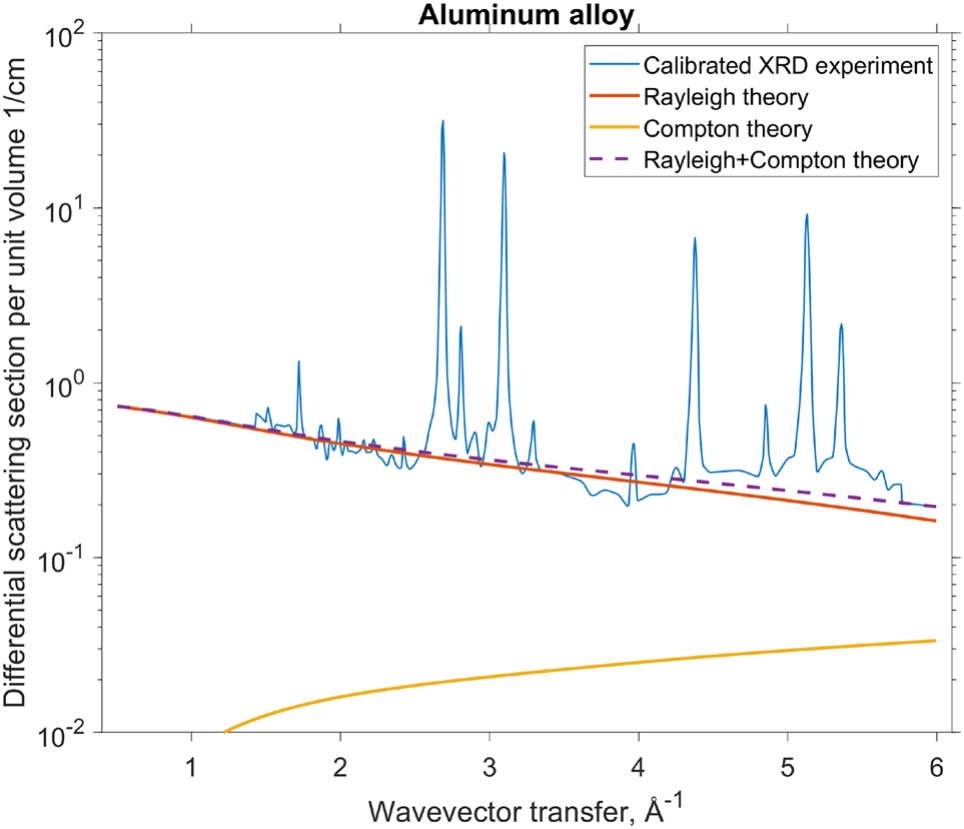
Experimental XRD pattern of an aluminum alloy^32^, rescaled by us to an absolute scale of $\hbox{cm}^{-1}$ to roughly match the theoretical, non-diffracting scattering, composed of Compton and Rayleigh terms. If experimental XRD data does not fully cover our desired range of 0.5–6Å$^{-1}$, we use the theoretical data for a non-diffracting material of the same composition.

Here $\rho$ is the mass density, $N_{1,2,\ldots }$ is the number of a given atomic species and $M_{1,2,\ldots }$ is the atomic weight. The classical electron radius is $r_e = 2.818\times 10^{-15}\,\hbox{m}$, while the Avogadro’s number is $N_A = 6.022\times ^{23}\,\hbox{mol}^{-1}$. Whereas Compton (incoherent) contribution is accurate for all materials, the Rayleigh (coherent) component would apply only if the atoms were distributed randomly in space. In real materials, the atomic structure is far from random, which results in constructive interference peaks and destructive interference valleys in the Rayleigh component. Roughly, the bottom of the measured XRD signal can reach as low as theoretical Compton scattering, while the baseline of the peaks (the inflection point) may roughly be where the theoretical Rayleigh $+$ Compton curve is. Using these rules-of-thumb we have the adjusted the amplitude of aluminum XRD data as shown in Fig. 6. We could also assess the validity of our rule-of-thumb in one case where the experimentally calibrated data was available (isotactic polypropylene^36^, not shown), and the two curves were well within the same order-of-magnitude.

As a side note, we mention that the polarization factor $\left( 1+\cos ^2\theta \right) /2$ is often not accounted for in published XRD data. Since the data is often collected using low energy X-rays, we have to divide it by its own polarization factor, before using the data in our simulations. This correction can be significant, up to a factor of 2, and is important to include because our XRD imaging scanner uses X-rays of much higher energy, hence the diffraction peaks at a given *q* value appear at substantially smaller scattering angles $\theta$.

For computing the ground truth (forward projection), the cross-section in Eq. (2) is obtained by smearing the high-resolution XRD data with the width of the scattering pathway from Eq. (32):

21 $$\begin{aligned} F(q) = \int _0^{\infty } \left( \frac{1}{V}\frac{d\sigma }{d\Omega }(q')\right) _{\text{XRD}} \frac{dq' }{\sqrt{2\pi \langle (\Delta q)^2 \rangle }} \exp\left(-\frac{(q' - q)^2}{2\langle (\Delta q)^2 \rangle }\right) \end{aligned}$$

### A small, but finite scattering pathway

**Figure 7:**
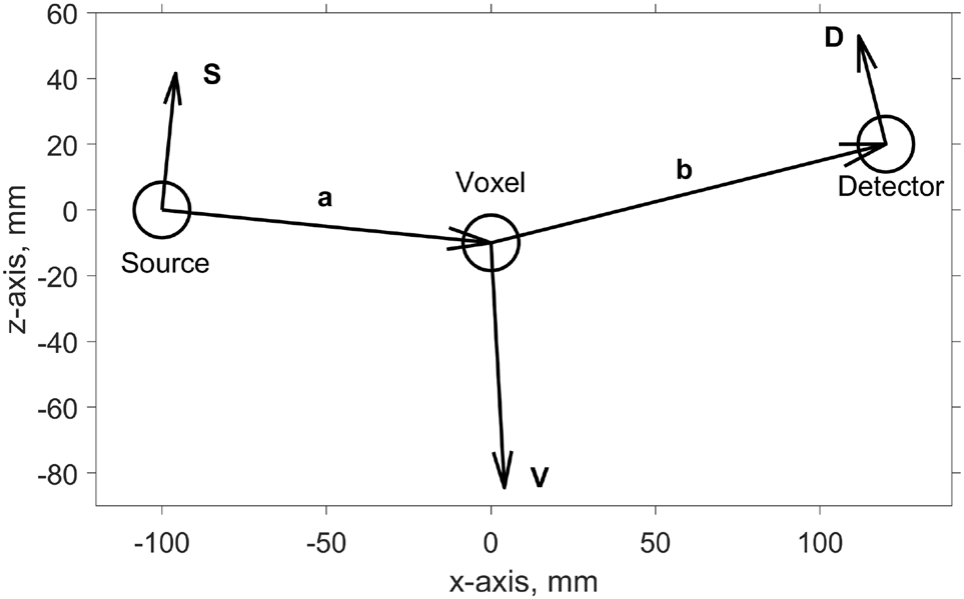
A typical scattering geometry, defined by the incoming ${\hat{\textbf{a}}}$ and outgoing ${\hat{\textbf{b}}}$ wave vectors. For the sake of generality, the two vectors are unequal. We also show the principal directions ${\textbf{S}}$, ${\textbf{V}}$, and ${\textbf{D}}$ which cause the main uncertainty $\Delta q$ (see Eq. (32)). The large circles illustrate the finite-sized source, voxel, and detector regions. Note that in general the three regions can have arbitrary, non-circular shapes as described in Eqs. (34)–(37). Specific points within those regions are labeled with vectors ${\textbf{s}}$, ${\textbf{v}}$, and ${\textbf{d}}$, respectively (not shown).

Diffraction patterns of high-quality crystals often have very sharp, narrow, peaks (see Fig. 6). To measure them, the experimental uncertainty of the wavevector transfer *q* should be substantially smaller than the intrinsic peak width. The *q*-resolution of our XRD tomography setup will be quite low compared to that of dedicated lab diffractometers, hence our reconstructed patterns will be smeared out. To quantify the smearing, in this chapter we derive the resolution formula for a realistic, finite-sized scattering pathway, as shown in Fig. 7. The accuracy of *q* is determined by the size of the source and the voxel, as well as the size and energy sensitivity of the detector.

The average wavevector transfer for a photon of energy *E*, traveling from the center of the source to the center of the voxel, and on to the center of the detector, is given by

22 $$\begin{aligned} {\textbf{q}} = \frac{E}{\hbar c}\left( {\hat{\textbf{b}}} - {\hat{\textbf{a}}}\right) \end{aligned}$$

Here $\hbar c= 1.973$ keV Å is the Planck’s constant multiplied by the speed of light. Since all three locations have a finite size, and the energy channel has a finite width, the wavevector transfer of each diffraction pathway has a finite distribution around the mean. We apply the chain rule to Eq. (22) to find the net change:

23 $$\begin{aligned} \Delta {\textbf{q}}= \frac{\partial {\textbf{q}}}{\partial E} \Delta E + \frac{E}{\hbar c} \left( \Delta {\hat{\textbf{b}}} - \Delta {\hat{\textbf{a}}}\right) = {\textbf{q}}\frac{e}{E} + \frac{E}{\hbar c} \left( \Delta {\hat{\textbf{b}}} - \Delta {\hat{\textbf{a}}}\right) \end{aligned}$$

Here we have used $e=\Delta E$ to label a small difference of X-ray energy with respect to the mean of the channel *E*. As for the geometrical spread, consider a scatterer located a small vector ${\textbf{v}}$ away from the center of the voxel. This will change the source-to-voxel vector to a new value

24 $$\begin{aligned} {\textbf{a}}' = {\textbf{a}} + {\textbf{v}} \end{aligned}$$

The difference of the incoming wave direction is thus

25a $$\begin{aligned} \Delta {\hat{\textbf{a}}}= \frac{{\textbf{a}}'}{a'} - \frac{{\textbf{a}}}{a} = \frac{{\textbf{a}} + {\textbf{v}}}{\sqrt{({\textbf{a}}+{\textbf{v}})^2}} - \frac{{\textbf{a}}}{\sqrt{{\textbf{a}}^2}} \end{aligned}$$

25b $$\begin{aligned}= \frac{1}{a} \left[ ({\textbf{a}}+{\textbf{v}}) \left( 1-\frac{{\textbf{a}}\cdot {\textbf{v}}}{a^2} + {\mathcal{O}} (v/a)^2\right) - {\textbf{a}}\right] \end{aligned}$$

25c $$\begin{aligned}= \frac{{\textbf{v}} - {\hat{\textbf{a}}} \left( {\hat{\textbf{a}}}\cdot {\textbf{v}}\right) }{a} + {\mathcal{O}} (v/a)^2 \end{aligned}$$

Neglecting higher order terms is well-justified when the voxel size *v* is much smaller than the source-to-voxel distance *a*. The linearized expression will be useful later on, in Eq. (32), where we compute the mean square width a scattering pathway. It would be exceedingly costly to compute the exact mean square width, as that involves a 10-dimensional integral (energy plus three points in 3D), for each possible diffraction pathway.

The next step in the derivation is to allow the source and the detector to have finite sizes too, described by small vectors ${\textbf{s}}$ and ${\textbf{d}}$ pointing away from their respective centers (the circles in Fig. 7). In other words, an arbitrary off-center scattering pathway is composed of two modified legs:

26a $$\begin{aligned} {\textbf{a}}'= {\textbf{a}} + ({\textbf{v}}-{\textbf{s}}) \end{aligned}$$

26b $$\begin{aligned} {\textbf{b}}'= {\textbf{b}} + ({\textbf{d}}-{\textbf{v}}) \end{aligned}$$

We then generalize Eq. (25) and combine it with Eq. (23) to find the full change of the wavevector transfer (accurate to first order in *s*, *v*, and *d*):

27 $$\begin{aligned} \Delta {\textbf{q}} = \frac{e}{E}{\textbf{q}} + \frac{E}{\hbar c} \left[ \frac{ ({\textbf{d}}-{\textbf{v}}) - \left[ {\hat{\textbf{b}}} \cdot ({\textbf{d}} - {\textbf{v}})\right] {\hat{\textbf{b}}}}{b} - \frac{ ({\textbf{v}}-{\textbf{s}}) - \left[ {\hat{\textbf{a}}} \cdot ({\textbf{v}} - {\textbf{s}})\right] {\hat{\textbf{a}}}}{a} \right] \end{aligned}$$

For isotropic materials, only the magnitude of the change is important:

28a $$\begin{aligned} \Delta q= \sqrt{({\textbf{q}}+\Delta {\textbf{q}})^2} - q \end{aligned}$$

28b $$\begin{aligned}= q \left[ 1 + \frac{{\textbf{q}}\cdot \Delta {\textbf{q}}}{q^2} + {\mathcal{O}}(\Delta q/q)^2\right] - q \end{aligned}$$

28c $$\begin{aligned}= \frac{{\textbf{q}}\cdot \Delta {\textbf{q}}}{q} + {\mathcal{O}}(\Delta q/q)^2 \end{aligned}$$

Neglecting terms of order $(\Delta q)^2$ and higher, the expression becomes

29a $$\begin{aligned} \Delta q= \frac{e}{E}q + \left( \frac{E}{\hbar c}\right) ^2 \left( \frac{{\textbf{s}}\cdot {\textbf{S}} + {\textbf{v}} \cdot {\textbf{V}} + {\textbf{d}}\cdot {\textbf{D}}}{qab}\right) \end{aligned}$$

29b $$\begin{aligned}= \frac{2e}{\hbar c}\sin (\theta /2) + \frac{E}{\hbar c} \left( \frac{{\textbf{s}}\cdot {\textbf{S}} + {\textbf{v}} \cdot {\textbf{V}} + {\textbf{d}}\cdot {\textbf{D}}}{2ab\sin (\theta /2)}\right) \end{aligned}$$

Here we have defined three auxiliary vectors

30a $$\begin{aligned} {\textbf{S}}= {\textbf{b}} - {\hat{\textbf{a}}} \left( {\hat{\textbf{a}}}\cdot {\textbf{b}} \right) \end{aligned}$$

30b $$\begin{aligned} {\textbf{D}}= -{\textbf{a}} + {\hat{\textbf{b}}} \left( {\hat{\textbf{b}}}\cdot {\textbf{a}} \right) \end{aligned}$$

30c $$\begin{aligned} {\textbf{V}}= -({\textbf{S}}+{\textbf{D}}) \end{aligned}$$

and used the amplitude of the mean wavevector transfer

31 $$\begin{aligned} q = |{\textbf{q}}| = \frac{E}{\hbar c} \sqrt{2\left( 1 - {\hat{\textbf{a}}}\cdot {\hat{\textbf{b}}}\right) } = \frac{2E}{\hbar c}\sin (\theta /2) \end{aligned}$$

The key quantity for computing realistic diffraction signals is the mean square width of a given scattering pathway:

32 $$\begin{aligned} \langle (\Delta q)^2 \rangle = \left( \frac{2\sin (\theta /2)}{\hbar c}\right) ^2\langle e^2 \rangle +\left( \frac{E}{\hbar c}\right) ^2 \left( \frac{\langle ({\textbf{s}}\cdot {\textbf{S}})^2\rangle + \langle ({\textbf{v}} \cdot {\textbf{V}})^2\rangle + \langle ({\textbf{d}}\cdot {\textbf{D}})^2\rangle }{[2ab\sin (\theta /2)]^2}\right) \end{aligned}$$

It contains four contributions, which is the range of energies, as well as non-zero sizes of the source, the voxel, and the detector. For sufficiently small bins, we can assume that the energy distribution within each energy bin is constant (uniform), resulting in

33 $$\begin{aligned} \langle e^2 \rangle = \frac{1}{\Delta E}\int _{-\Delta E/2}^{\Delta E/2} e^2\; de = \frac{(\Delta E)^2}{12} \end{aligned}$$

The size of the source refers to the spatial distribution of points that emit X-rays. In general, it is a 3D scalar function $\rho ({\textbf{s}})$ (assumed to be normalized $\int \rho ({\textbf{s}})\; d{\textbf{s}} = 1$) and the relevant moment for us is

34 $$\begin{aligned} \langle ({\textbf{s}}\cdot {\textbf{S}})^2 \rangle = \int \rho ({\textbf{s}}) ({\textbf{s}}\cdot {\textbf{S}})^2\; d{\textbf{s}} \end{aligned}$$

In this paper we do not investigate the detailed shape of $\rho ({\textbf{s}})$, and instead assume a simplified model, namely a flat square patch of the anode surface area whose normal vector is given by ${\hat{\textbf{n}}}$. In this case, Eq. (33) generalizes to

35 $$\begin{aligned} \langle ({\textbf{s}}\cdot {\textbf{S}})^2 \rangle = \frac{(\Delta s)^2}{12} \left( {\textbf{S}}^2 - ({\textbf{S}}\cdot {\hat{\textbf{n}}})^2 \right) = \frac{(\Delta s)^2}{12} |{\textbf{S}}\times {\hat{\textbf{n}}}|^2 \end{aligned}$$

where $\Delta s = 0.5\,\hbox{mm}$ is the side length of the rectangular patch that emits X-rays, a.k.a the focal spot. The same formula also applies for the detector, which we set to be a rectangle of surface area $|{\textbf{A}}| = 0.5\,\hbox{mm}^2$:

36 $$\begin{aligned} \langle ({\textbf{d}}\cdot {\textbf{D}})^2 \rangle = \frac{|{\textbf{D}}\times {\textbf{A}}|^2}{12A} \end{aligned}$$

Lastly, the voxel is modeled as a rectangular parallelepiped of uniform density, in which case the formula generalizes to

37 $$\begin{aligned} \langle ({\textbf{v}}\cdot {\textbf{V}})^2 \rangle = \frac{(\Delta x V_x)^2 + (\Delta y V_y)^2 + (\Delta z V_z)^2}{12} \end{aligned}$$

### The forward projector and model matrix

We now have all the ingredients to carry out the calculation for the expected number of photons, Eq. (2). To perform the inverse calculation, we first have to discretize the *q*-axis to NWT finite-sized bins. Generally, the bins should be somewhat smaller than the intrinsic, physical scanner resolution $\sqrt{\langle \Delta q^2\rangle }$. It is futile to attempt reconstruction at a much finer resolution than that, especially because the matrix size and computational time are already strained and require excessive effort to compute in practice. In this project, we work with $\text{NWT} = 256$ reconstruction bins, spaced unequally to roughly follow the natural behavior of $\sqrt{\langle \Delta q^2\rangle }$. Furthermore, to speed up calculations, we set the diffraction signal to zero outside of the range $q_{\text{min}} = 0.5$ Å^−1^ and $q_{\text{max}} = {6.0}$ Å^−1^ in both forward and inverse problems. In reality, a small amount of diffracted photons may reach the detector beyond this range, and we could address it as a general background bias as shown in Eq. (1), but this is beyond the scope of the current work. We define the left edge of our reconstruction bin nwt to be

38 $$\begin{aligned} q_{\text{left}}(\text{nwt}) = q_{\text{min}} + {\text{nwt}}*dq_0 + (q_{\text{max}} - q_{\text{min}} - dq_0*{\text{NWT}})\frac{\text{nwt}^2}{\text{NWT}^2} \end{aligned}$$

where $dq_0 = 0.01$ Å^−1^ is the width of the first bin. The right edge $q_{\text{right}}(\text{nwt})$ is the same but with $\hbox{nwt}+1$ instead of nwt. The average wavevector of the bin is

39 $$\begin{aligned} q_{\text{bin}}(\text{nwt}) = \frac{q_{\text{left}}(\text{nwt}) + q_{\text{right}}(\text{nwt})}{2} \end{aligned}$$

The (*m*,*k*)-th element of the matrix ${\mathbb{A}}$ is the sum of intersections of the reconstruction bin and each physical pathway, namely

40 $$\begin{aligned} {\mathbb{A}}(m,k) = \sum _{\text{vox}=1}^{\text{VOX}} G \sum _{\text{nrgsrc}=1}^{\text{NRG}} \left( \frac{q_{\text{right}} - q_{\text{left}}}{\sqrt{2\pi \langle (\Delta q)^2 \rangle }}\right) \exp \left( -\frac{(q_{\text{bin}} - q)^2}{2\langle (\Delta q)^2 \rangle } \right) \eta P_{\text{in}} P_{\text{out}} \end{aligned}$$

Notice how the above equation contains every aspect of the scan, except the diffraction pattern *F*. To verify the validity of the matrix, we can multiply it with a column vector (see Eq. (1)) made from bin-averaged scattering cross-sections:

41 $$\begin{aligned} F(k) = \int _{q_{\text{left}}}^{q_{\text{right}}} \frac{dq'}{q_{\text{right}} - q_{\text{left}}}\left( \frac{1}{V}\frac{d\sigma }{d\Omega }(q')\right) _{\text{XRD}} \end{aligned}$$

The resulting photon counts *N*(*m*) are close to what we get from direct ground truth summation (Eq. (2)), with small differences due to the finite size of discretization (not shown).

### Compton scattering background

**Figure 8:**
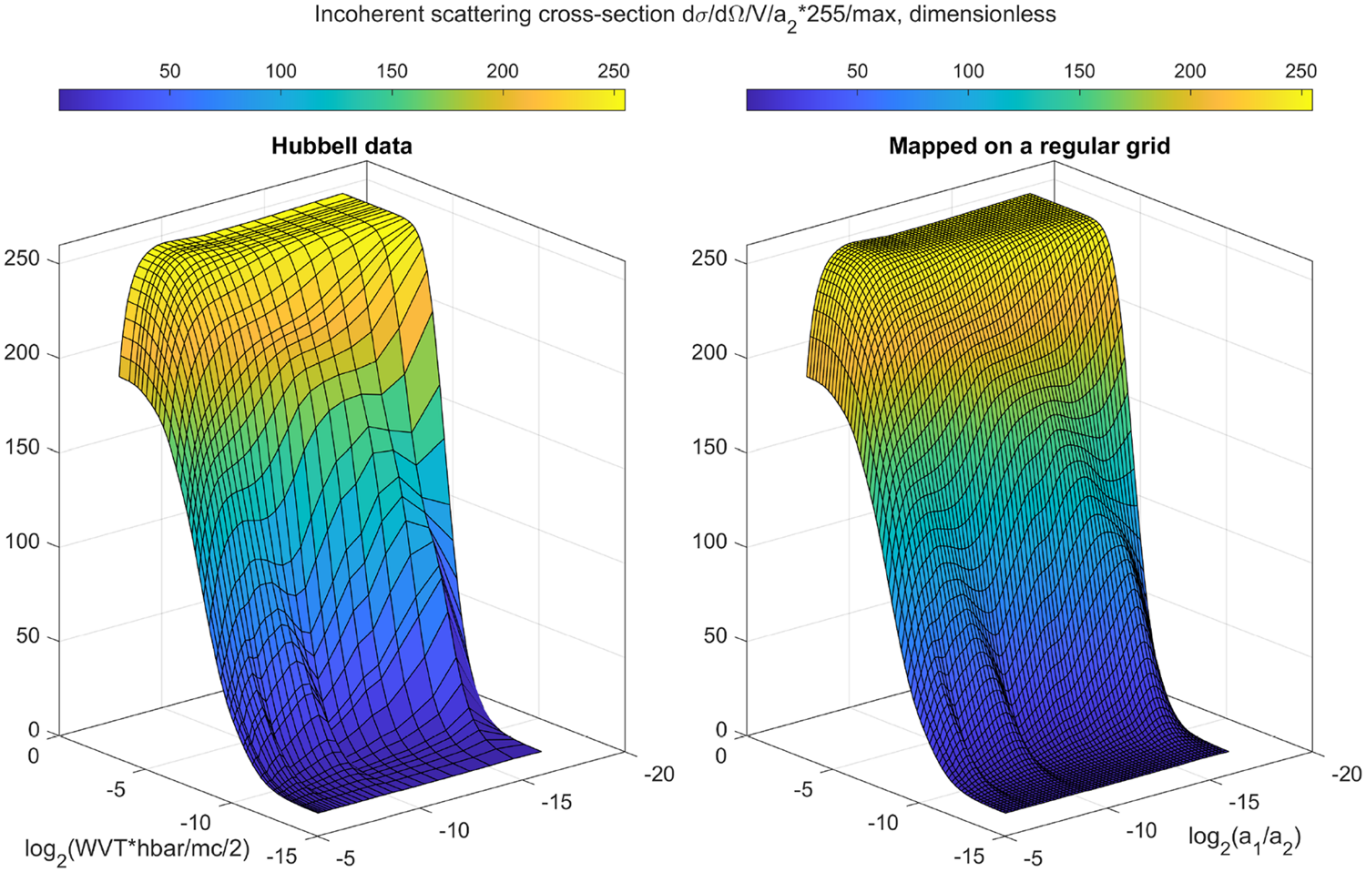
The map from MECT output ($a_1$, $a_2$) to the Compton scattering cross-section. The wavevector transfer is expressed in dimensionless units $q\hbar /mc/2$. Both inputs are on logarithmic axis of base 2 for quicker lookup using a regular 64x64 grid (right), obtained by interpolating Hubbell’s irregularly spaced data (left), available in Ref.^37^. The lookup table is normalized to the range [0, 255], so it can be stored as 8 bit integers, to reduce critical shared memory usage.

There can be many sources of bias in Eq. eqrefmatrixproduct, due to imperfect instrumentation (detector blur, lag), as well as secondary X-ray phenomena like scattering. Here we consider bias due to incoherent (Compton) scattering, which may become significant at higher energies and scattering angles. Only a small addition to our previously described XRD calculation is needed to obtain the single-scattered Compton signal as well. Multiple scattering (both coherent, incoherent, and combinations thereof) is beyond the scope of this work, although some progress has been reported in literature in the context of XRD tomography^38^. The number of Compton-scattered photons is computed similarly as in Eq. (2), except that the scattering cross-section *F* is replaced by the one interpolated from Hubbell’s tables, see Fig. 8. It depends on the photoelectric and Compton coefficients $a_1$ and $a_2$ of each material, which we have already assumed will be available from transmission MECT. We also see that the incoherent scattering cross-section varies smoothly with *q*, hence we do not need to pre-average it (i.e., we can assume that it is constant over the width of the scattering pathway $\sqrt{\langle (\Delta q)^2 \rangle }$). However, keep in mind that Compton scattering is inelastic, meaning that the electron carries away some of the momentum, which changes the wavevector transfer of the photon:

42 $$\begin{aligned} \frac{\hbar q_{\text{Compton}}}{2mc} = \left( \frac{\hbar q}{2mc}\right) \left( \frac{1}{\epsilon }\right) \sqrt{\epsilon + \left( \frac{\hbar q}{2mc}\right) ^2} \end{aligned}$$

The above equation is in dimensionless units, and is used to fetch tabulated cross-section data as shown in Fig. 8. The symbol $\epsilon$ is short for

43 $$\begin{aligned} \epsilon = 1+(1-\cos \theta )E_{\text{in}}/mc^2 \end{aligned}$$

which is also used to calculate the energy of the outgoing photon:

44 $$\begin{aligned} E_{\text{out}} = \frac{E_{\text{in}}}{\epsilon } \end{aligned}$$

We apply the above equation to the left and the right edge of the incoming source photon energy bin, resulting in the two edges of the outgoing energy band, $E_{\text{out1}}$ and $E_{\text{out2}}$. We then overlap this range with the detector energy channel range:

45 $$\begin{aligned} \text{overlap} = \frac{ \text{max}[0, \text{min}(E_{\text{out2}}, E_{\text{det2}}) - \text{max}(E_{\text{out1}}, E_{\text{det1}})]}{E_{\text{out2}}-E_{\text{out1}}} \end{aligned}$$

The dimensionless overlap weight above is multiplied with all the terms that enter the sum in Eq. (2). In other words, we perform accumulation with linear interpolation. A simpler, nearest-neighbor approach is also possible, but since our XRD summation code already has all the detector energy channels, we reuse the program structure for Compton as well. Lastly, for Compton scattering, the polarization factor in Eq. (7) has to be replaced by the Klein–Nishina function:

46 $$\begin{aligned} {\text{Klein}}{-}{\text{Nishina}} = \epsilon ^2\left( \frac{\epsilon + \epsilon ^{-1} - 1 + \cos ^2\theta }{2}\right) \end{aligned}$$

where $\epsilon = E_{\text{in}}/E_{\text{out}}\ge 1$ is the ratio of the incoming to outgoing energies. In the limit of small energy and/or scattering angle, the Klein–Nishina factor reduces back to the polarization factor $\left( 1+\cos ^2\theta \right) /2$.

## Results

Our example phantom (Fig. 1) contains three large bodies made from low density cellulose (akin to clothes), and wrapped inside we have a few diffracting objects, one benign (aluminum alloy) and two threats (both ammonium nitrate). All objects are fairly large and round, hence the transmission MECT data should be very good, allowing for an accurate image segmentation, which we assume is known. The ground truth is obtained by digitizing high-resolution experimentally measured XRD data available in literature. We also require the density and chemical composition of each material in order to simulate the attenuation (see “Beam attenuation” section). We have used these literature sources:

- Cellulose is taken from Ref.^39^, and we assume density of 0.1g/cm$^{3}$ to be a reasonable model for clothes.
- Aluminum alloy is taken from Ref.^32^, using the composition listed in the same paper.
- Ammonium nitrate is taken from Ref.^40^. It is a hazardous material, commonly used as fertilizer, but can be easily detonated, whether intentionally or not.

We have calibrated the above data on an absolute scale as explained in “Diffraction patterns” section, which produces the differential scattering cross-section per unit volume (without the polarization factor): $V^{-1}(d\sigma /d\Omega )_{\text{XRD}}$.

**Figure 9:**
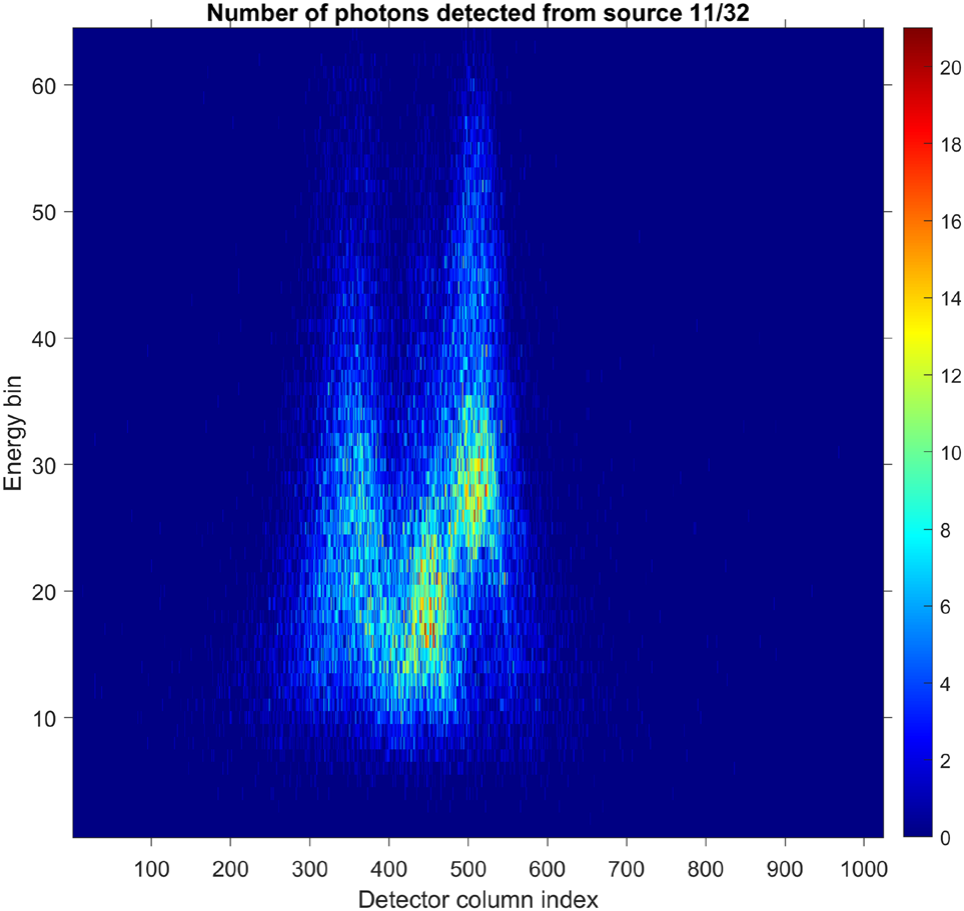
One slice of the deterministic forward simulation, with Poisson noise added as described in Eq. (1). In total there are approximately 44,000 photons in this detector snapshot, or about 1,100,000 photons for all 32 source positions.

**Figure 10:**
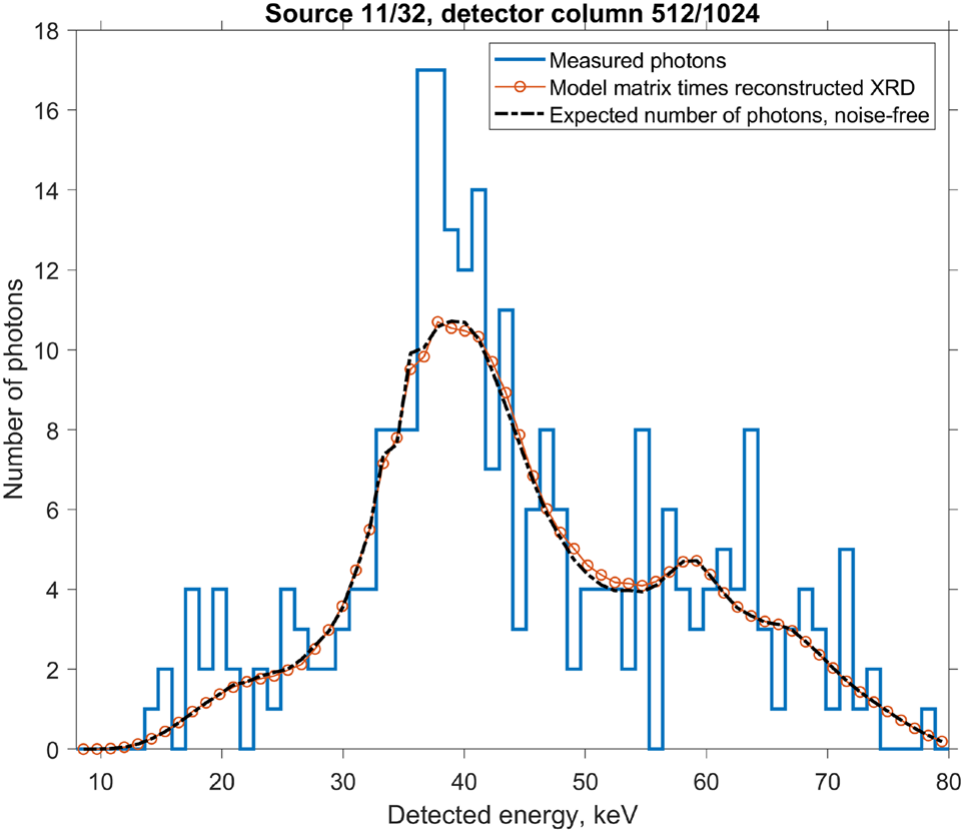
One-dimensional slice of the noisy measurement from Fig. 9. For comparison, we show the number of photons ${\textbf{N}} = {\mathbb{A}}\cdot {\textbf{F}}+{\text{bias}}$, computed using the reconstructed ${\textbf{F}}$, as well as the noise-free ground truth calculation for the number of photons, Eq. (2).

Since the resolution of laboratory XRD from literature far exceeds that of our proposed airport scanner, we must smear the ground truth over the width $\langle (\Delta q)^2\rangle$ of each individual diffraction pathway as shown in Eq. (21). We then apply Eq. (2) to compute the expected number of XRD photons for each measurement. We also add the incoherent Compton scattering as explained in Section refsec:compton. Lastly, we use a Poisson random number generator to simulate photon counting noise as shown in Eq. eqrefmatrixproduct. One 2D slice of this forward simulation is shown in 2D in Fig. 9, and a 1D slice is shown in Fig. 10 (the blue staircase).

**Figure 11:**
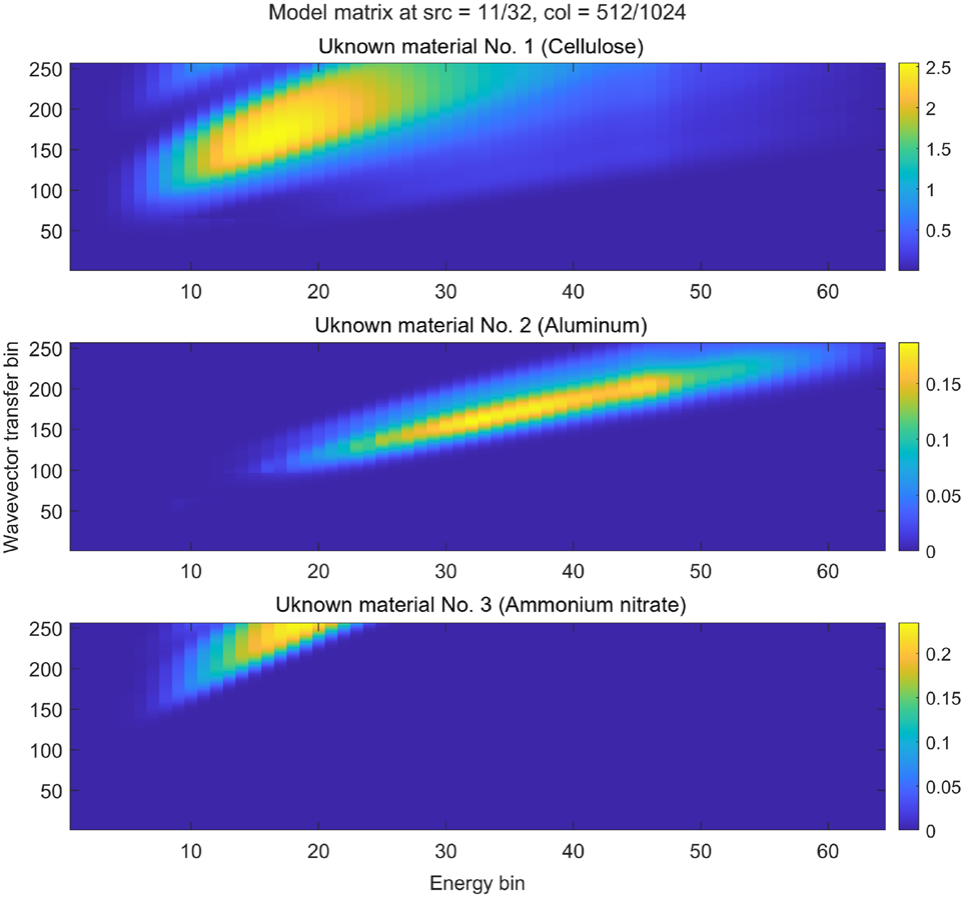
Showing one 3D cross-section of the full 5D matrix ${\mathbb{A}}$. Notice that these coefficients also depend on the remaining two dimensions, i.e., the source-detector pair (only a single pair is shown here). In particular, there are more non-zero weights beyond 30 keV for the unknown material No. 3 at other pairs, all of which are used in the reconstruction. The matrix ${\mathbb{A}}$ does not depend on the nature of the materials. The identities of the three unknown materials will only be revealed after reconstruction and fingerprinting against the database of known diffraction patterns.

To perform XRD reconstruction (Step 4 in the imaging workflow), we first compute the model matrix ${\mathbb{A}}$ using Eq. (40). The result (one slice) is shown in Fig. 11. This computation requires a prior MECT reconstruction, which provides the photoelectric and Compton coefficients $(a_1, a_2)$ at a high spatial resolution (see Fig. 1). In addition, we use $(a_1,a_2)$ to simulate Rayleigh and Compton scattering that is expected to fall on the XRD detectors (see Fig. 8). The simulated Compton contribution is used to correct for bias, while the Rayleigh contribution can be used as a starting point ${\textbf{F}}^{(0)}$ of an iterative reconstruction scheme. Here we apply the Lucy–Richardson algorithm, which although not always fast, is robust against strong Poisson noise:

47 $$\begin{aligned} {\textbf{F}}^{(i+1)} = \left( \frac{{\textbf{F}}^{(i)}}{\sum _{m=1}^M{\mathbb{A}}(m,k)} \right) * {\mathbb{A}}^T\cdot \left( \frac{{\textbf{N}}}{{\mathbb{A}}\cdot {\textbf{F}}^{(i)} + \text{bias}}\right) \end{aligned}$$

In the above equation, the star ($*$) symbol and the divisions are point-wise operations, while the dot ($\cdot$) symbol is the matrix-vector product. The bias in the denominator is the simulated Compton scattering, which is always positive, so we never have a zero in the denominator. The algorithm is multiplicative which guarantees that the solution stays non-negative. Each iteration is guaranteed to reduce the Poisson likelihood cost function, which is appropriate for very low photon counts. Typically, the solution converges within a few hundred iterations, and that is much faster than building the matrix ${\mathbb{A}}$ itself.

**Figure 12:**
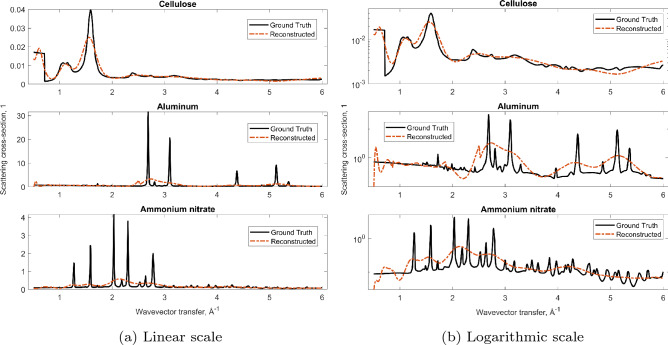
Reconstructed vs ground truth diffraction patterns. Cellulose is an amorphous material, hence its diffraction peaks are very broad and thus easily measured with our low resolution XRD tomography. The other two materials are quite crystalline, about 10x above the resolution of our example scanner. Nevertheless, the three substances are still clearly distinguishable. In particular, one material is consistent with a known threat (ammonium nitrate). The suitcase can thus be flagged for manual search. Other benign materials could also have given rise to a similar reconstruction (a false positive). However, the scanner is still a useful screening tool as it reduces the need to manually search suitcases where none of the reconstructed patterns are consistent with known threats. The rate of false positives can be reduced by e.g., narrowing the slit opening, Eq. (37), which will increase the resolution, but lower the flux, hence the screening time is prolonged.

The last step in the XRD imaging workflow is fingerprinting the reconstructed diffraction patterns against a database of known materials (Step 5). Building such a database is a future project. For now, we only compare our reconstruction results against the initial ground truth, shown on linear and logarithmic scales (Fig. 12). As anticipated, each reconstructed curve is essentially a low-resolution version of the ground truth. For now we only inspect it visually on linear and logarithmic scales, and observe a qualitative match. We have not developed a figure-of-merit (FOM) to quantify the degree to which a reconstructed pattern matches a high-resolution pattern from a database. A naïve FOM like the sum of square errors (on either linear or logarithmic scales) is likely not robust enough for a real scanner. For example, there may be a calibration error that shifts the reconstructed pattern along the *q*-axis, in which case the squared error is high, even though the overall pattern has the correct shape. A more robust FOM could be Earth Mover’s Distance (Ref.^41^), or even better a 1D deep neural net (Ref.^42^) trained on real suitcases.

Fingerprinting aside, we perform a sanity check on the result of Eq. (47). We plug the reconstructed ${\textbf{F}}$ back into Eq. (1), and plot the resulting product as red circles in Fig. 10. As expected, the outcome is very similar to the noise-free forward model (black dashed curve). To summarize, in this section we have (1) performed a forward simulation using literature ground truth data, (2) applied Poisson noise, (3) inverted the noisy measurement using the Lucy–Richardson algorithm, (4) forward-projected the result and verified that it matches the initial simulation in step 1. The reconstructed diffraction patterns resemble the ground truth, but have a lower resolution, as expected.

## Discussion

We have started this article with a literature overview, “Literature overview” section, that includes dozens of experimental and real-world applications for XRD tomography. There is no doubt that the idea of reconstructing multiple XRD patterns from large phantoms is valid and works in practice. It is also clear from the literature, as well as intuitively, that photon flux is higher for geometries with less collimation. However, note that the relationship between flux and reconstruction quality is not linear. From Poisson statistics^43^, we know that reconstruction error due to shot noise is proportional to the inverse square of the number of detected photons, i.e., $1/\sqrt{N}$. This function is fairly flat when *N* is high, but it increases very steeply as $N\rightarrow 0$. As a consequence, in case of very low flux, every photon is precious and increasing the flux even slightly will result in a significantly better measurement.

Our main contribution was to show how to reconstruct XRD patterns from a minimally collimated scan. The numerical quality of our results is very good, as evidenced in Fig. 10 by a match between the forward projection of the ground truth (black dashed curve) and the forward projection of the reconstructed data (red circles). Of course, in reality, there will be many artifacts that will degrade the reconstruction quality. In particular, our key equation, Eq. (32), contains simplified theoretical inputs for the sizes of the focal spot, voxel thickness, and the detector pixel. Also, Eq. (12) uses a theoretical SpekCalc simulation for the source spectrum, and assumes a perfect detector efficiency. In reality, inputs like these will have to be experimentally calibrated for any given scanner, although the functional form of our equations should remain valid.

The resolution of our reconstructed XRD patterns is primarily limited by the energy sensitivity of available X-ray detectors, see Eq. (11). This hardware parameter is outside of our control, although the technology has room to improve, especially if a large market like airport security starts demanding it. Another key parameter that we can control is the slit opening $\varphi _1-\varphi _2$, which determines the voxel thickness, Eq. (8). This opening is directly proportional to photon flux, but it also degrades the resolution, Eq. (32), since the auxiliary vector ${\textbf{V}}$ has a large component along the *z*-axis, see Fig. 7. Typical MECT scanners have an opening of 1-2$^\circ$, while typical XRD machines have a beam divergence closer to $0.02^{\circ }$^44^. Here we have assumed a reasonable middle ground of 0.5$^\circ$, although this parameter will have to be further tuned with a real scanner.

The reconstruction algorithm that we have used, Eq. (47), is fairly generic, and may be far from optimal considering the unique challenges of XRD imaging. In particular, our model is a linear system of ${\mathcal{O}}(10^6)$ equations (one for each relevant source-detector-energy bin) and ${\mathcal{O}}(10^3)$ unknowns, which are the handful of one-dimensional diffraction patterns. At face value this looks like a highly overdetermined system, hence it should be easy to solve. However, the information contained in each equation is strongly overlapping. This redundancy stems from the intrinsically low resolution. Furthermore, the photon counts can be very low, resulting in many measurements with zero counts (the output is sparse). Lastly, the diffraction patterns are not free to assume just any random shape, so out of the ${\mathcal{O}}(10^3)$ unknowns perhaps just ${\mathcal{O}}(10)$ are truly independent. To sum up, we need to reconstruct a dozen of degrees of freedom from a million measurements, while fully anticipating that those measurements will have poor resolution and high noise. It is a complex problem and could benefit from more advanced reconstruction techniques, including

- edge-preserving and detail-preserving regularization,
- enforcing sparsity in a suitable basis (e.g., in a wavelet basis),
- searching for diffraction patterns from a low-dimensional functional space (e.g., a smooth background plus a few spikes with unknown locations and heights),
- deep learning, see Refs.^45–48^.

Many of the challenges that we have encountered are unique to airport security. It is a particularly demanding area for X-ray imaging, because of the enormous variability in the size, shape, and composition of materials found in suitcases. On the flip side, there is much interest in XRD tomography for domains other than airport security, such as healthcare or manufacturing quality control. Examples include imaging defects in concrete^49^ and metals^50^, especially for quality control in 3D printing^51^. Whereas transmission tomography has a resolution of ${\mathcal{O}}(10^{-3}\,\hbox{m})$, diffraction tomography can reveal details in the ${\mathcal{O}}(10^{-9}\,\hbox{m})$ range. The nanostructure of a material can be inferred by fitting a molecular model to the reconstructed diffraction pattern. For example, if we measure a diffraction peak at $q=1$ Å^−1^, Bragg’s law says that it corresponds to a lattice of atoms with a spacing of $d=2\pi /q = 0.63\,\hbox{nm}$. Such information has been used to reveal the nanostructure of bones^52^ and calcifications in soft tissues like human breast^53^.

In certain applications like those mentioned above, there are only a small number of diffracting materials, often known beforehand. In addition, the MECT modality provides all the spatial information (locations, shapes of objects), as well as some information on material composition (density and average atomic number). Very often, the purpose of an X-ray scan is to answer a simple yes/no question (threat vs benign, cancer vs healthy, etc.). If that is the case, there may be no need to run the costly and error-prone XRD inversion, Eq. (47). It could be much faster and more robust to just run forward simulations using Eq. (2) with a list of known diffraction patterns ${\textbf{F}}$. We then only need to check which among the possible sets of materials gives rise to data closest to the measurement ${\textbf{N}}$. Even in the difficult case of airport security, there are no more than a few dozen kinds of explosives and a handful of drug types that are commonly smuggled. If we could cost-effectively increase the detection accuracy for just a few of the most common threats, that would aid law enforcement tremendously.

## Conclusion

In this work we have shown how to identify multiple materials from a large phantom using XRD tomography. The setup is essentially the same as fan-beam MECT, but with an additional detector on one (or both) sides of the fan. Only diffracted photons can reach those areas, and without a detector, the information carried by them would be lost. In this design there are no collimators beyond those required by fan-beam MECT, resulting in the highest possible XRD photon flux. With transmission alone, MECT can only yield two numbers per voxel (the photoelectric and Compton coefficients). Our reconstruction, while far from the quality of small sample laboratory XRD, gives much more information than just two numbers, see Fig. 12.

In conclusion, an XRD imaging add-on to existing CT scanners is feasible and is well-positioned to provide unique, material-specific information, at a low cost of installing an extra detector or two, and developing suitable reconstruction software.

## Data Availability

The datasets generated during the current study are not publicly available due to legal restrictions but are available from the corresponding author on reasonable request.
